# Metadata management for high content screening in OMERO

**DOI:** 10.1016/j.ymeth.2015.10.006

**Published:** 2016-03-01

**Authors:** Simon Li, Sébastien Besson, Colin Blackburn, Mark Carroll, Richard K. Ferguson, Helen Flynn, Kenneth Gillen, Roger Leigh, Dominik Lindner, Melissa Linkert, William J. Moore, Balaji Ramalingam, Emil Rozbicki, Gabriella Rustici, Aleksandra Tarkowska, Petr Walczysko, Eleanor Williams, Chris Allan, Jean-Marie Burel, Josh Moore, Jason R. Swedlow

**Affiliations:** a Centre for Gene Regulation & Expression, University of Dundee, Dundee, Scotland, UK; b Glencoe Software, Inc., Seattle, WA, USA

**Keywords:** Data management, Screening, Metadata, HCS

## Abstract

High content screening (HCS) experiments create a classic data management challenge—multiple, large sets of heterogeneous structured and unstructured data, that must be integrated and linked to produce a set of “final” results. These different data include images, reagents, protocols, analytic output, and phenotypes, all of which must be stored, linked and made accessible for users, scientists, collaborators and where appropriate the wider community. The OME Consortium has built several open source tools for managing, linking and sharing these different types of data. The OME Data Model is a metadata specification that supports the image data and metadata recorded in HCS experiments. Bio-Formats is a Java library that reads recorded image data and metadata and includes support for several HCS screening systems. OMERO is an enterprise data management application that integrates image data, experimental and analytic metadata and makes them accessible for visualization, mining, sharing and downstream analysis. We discuss how Bio-Formats and OMERO handle these different data types, and how they can be used to integrate, link and share HCS experiments in facilities and public data repositories. OME specifications and software are open source and are available at https://www.openmicroscopy.org.

## Highlights

• HCS data management is challenging due to its scale, complexity and heterogeneity.
• OMERO and Bio-Formats are open-source tools for data access and management at scale.
• OMERO and Bio-Formats can handle images, experimental metadata and analytic outputs.
• Repositories integrating multiple image-based studies provide tests of the value of data integration.

## 1 Introduction

High content screening (HCS) experiments inevitably combine several types of experimental information that must be linked, integrated, and processed into a set of interpretable results, shareable in a report or scientific paper. These related, but distinct sets of data—experimental metadata describing protocols, reagents and data acquisition; images recording the structure and dynamics of the cells and/or tissues being assayed; and downstream analytic output converting image-derived phenotypes into qualitative or quantitative metadata—all comprise a single “experiment” or “assay”. They must be linked and integrated to enable understanding and interpretation of an HCS experiment. Data management functions—software tools that deliver data linkage and integration—are therefore a critical component of HCS experiments.

In many scientific applications, data management is implemented using a file-based approach. Experimental metadata, binary data (in HCS experiments, this is the image data) and analytic metadata are stored in files on a filesystem. Experimental and analytic metadata stored in spreadsheets is relatively simple to read and write, and linkages to image data files (names and locations of files, etc.) can be stored alongside metadata. This approach is used quite often in small labs—it is simple to implement and easy to understand. However, as data volumes grow in size and complexity, more sophisticated systems are required to query, process and access complex, highly integrated and linked datasets. Metadata must be stored in a database that allows querying and processing by large, distributed computational resources. In many cases, coordinated access to metadata and binary data are necessary.

Since 2000, the Open Microscopy Environment (OME) has been building and releasing open specifications and software that provide data management resources for biological and biomedical imaging. OME has three components—an open data model and file formats for biological imaging (OME Data Model and OME-TIFF), software libraries for data file conversion (Bio-Formats), and software tools for image data management and analysis (OMERO). In 2008, we presented our first overview of using OMERO for HCS data [1]. In this paper, we present an update on the usage of Bio-Formats and OMERO for HCS data, and focus on the latest strategies available in OMERO for storing and managing the many different types of metadata recorded and used in modern HCS experiments.

## 2 Methods

### 2.1 Software

Bio-Formats is developed and released in Java, with single jar files available for download (https://downloads.openmicroscopy.org). The software is built by reverse engineering datasets submitted by the scientific community. Once a reader for a specific file format is built, it is tested daily against submitted files (https://ci.openmicroscopy.org). As of this writing (July 2015), >31,000 datasets made up of >600,000 files totaling >5.2 TB are used for developing and testing Bio-Formats. A detailed description of the design and architecture of Bio-Formats has been published [2].

OMERO is an enterprise data management application that combines mechanisms for storing and accessing image metadata, binary pixel data, text-based tag and file annotations, and analytic output [3]. OMERO is built as a Java-based middleware application that links a PostgreSQL relational database, a Lucene-based search index, a filesystem-based image repository and an HDF-based tabular data store [3]. OMERO’s client–server architecture enables remote access to the data it holds. OMERO’s permissions system controls access to that data ensuring that each dataset, image, annotation or analytic result is only retrievable by those with correct permissions to do so [4]. A Python-based scripting engine provides an interface for data processing and supports processing and analysis applications. Several examples of integrating analysis tools into OMERO have been published [3], [5], [6], [7].

Bio-Formats and OMERO are built upon the OME Data Model, a specification for metadata related to imaging [8], [9]. They use the OME Data Model to natively support 5D imaging (space, time and channel) [10] and have extension points for reagents, multiple illumination paths (e.g., fluorescence recovery after photobleaching (FRAP), or photo activation or photoconversion), and specialised multi-dimensional imaging modalities like fluorescence lifetime imaging (FLIM) and optical projection tomography (OPT) [5].

This model-based approach allows Bio-Formats and OMERO to progressively support new metadata types and imaging domains, without a complete re-engineering of the software. OMERO uses the Ice library (http://zeroc.com) to provide an application programming interface (API) that supports client environments built in HTML, Python, Java, C++, and several frameworks, including Matlab. With Bio-Formats providing access to >140 image file formats [2] and OMERO providing support for most major data visualisation and processing environments, this platform provides access for most modern software tools and imaging modalities in use in the life and biomedical sciences. OMERO has been recently updated (Feb 2014) to read data directly from image data files in their proprietary file formats using a substantially enhanced Bio-Formats library [11].

### 2.2 Process

The OME codebase is stored and accessed on Github (https://github.com/openmicroscopy). Code fixes, updates and new functions are submitted by a member of the OME team or the wider community and then reviewed by another member of the team (https://www.openmicroscopy.org/site/support/contributing/). If approved, they are checked for adherence to code style and formatting guidelines by an automatic tool (SCC; (https://github.com/openmicroscopy/snoopycrimecop)), merged with the rest of the code base and automatically run through a series of tests using OME’s continuous integration system (https://ci.openmicroscopy.org). Any failing tests are reported and corrected. In preparation for a release, the software is manually run through a series of testing scenarios that exercise most of the known use cases and user workflows. Once all tests pass, the software is released for download (https://downloads.openmicroscopy.org).

## 3 Results

### 3.1 Data import and access: OMERO and Bio-Formats 5

Starting with OMERO 5.0 (released February 2014) we have implemented a new approach for data access. From this release forward, image data are read directly from native files via Bio-Formats. The OMERO.server is connected to a filesystem containing image data and all relevant metadata is imported into OMERO’s database. Access to binary image data is achieved in real time by using Bio-Formats to read pixel data directly from the original image data formats. This approach substantially accelerates data import—it eliminates lengthy transfers of large binary pixel data into OMERO and prevents unnecessary data duplication. The technical details of this new data access strategy have been recently published [11].

For HCS data—large datasets comprised of 10^3^–10^6^ individual images—this approach substantially improves OMERO’s performance and utility. In addition, OMERO 5’s import strategy also allows users and sysadmins to access data from multiple sources, thereby providing more flexibility and adaptability to individual institutions’ storage strategies.

In Bio-Formats and OMERO 5.1 (released April 2015), we again delivered on improved performance for Bio-Formats, especially for networked file systems. We reduced the overhead of file opening and improved caching of image metadata in Bio-Formats. For production data acquisition facilities, we also expanded the ability for one user (e.g., a facility manager) to import data for another (e.g., a scientist user of the facility’s resources).

These changes substantially improve and enhance OMERO’s performance and utility for large-scale data processing. For calculations distributed across a multi-node cluster, access to metadata and annotations is achieved through the OMERO API, whereas access to binary image data is achieved directly using Bio-Formats, from image data files stored on a clustered file system (e.g., GPFS, Lustre, etc.). This flexibility is important regardless of the size or complexity of the image processing calculations. Even simple calculations can be a major challenge for an HCS data management system. Examples are the calculation of thumbnails or of basic image metadata parameters (minimum, maximum, mean, median intensity), and the re-calculation of thumbnails with new rendering settings. Since each image has to be read before a thumbnail can be calculated, performance is limited by access to binary image data. For these large calculations, the OMERO API can be used for metadata and result handling capabilities (see below, 3.3) and a distributed calculation can take advantage of an appropriate filesystem to avoid I/O bottlenecks. The same concept applies for more complex calculations like object segmentation or multi-parametric feature calculation that also depend on access to binary image data.

### 3.2 HCS data sharing and publication

Most scientific enterprises have a critical need to securely share large datasets between colleagues or collaborators, regardless of location. In some cases, data sharing may be limited to read-only access, where data can only be viewed. In other cases, full interactive access is required, for example, where data collected in an imaging facility is analysed or mined by a collaborator or other resource. Finally, some subsets of data, including specific annotations or analytic results may be published on-line for public access. OMERO supports all these use cases, even for large HCS datasets. A detailed description of OMERO’s data sharing and publication facilities has been published recently [4].

### 3.3 Metadata management for HCS datasets

In HCS experiments, the term “metadata” refers to different types of experimental parameters and analytic outputs. These take different forms, are on different scales and are used for different purposes. For this reason, OMERO provides multiple ways of storing, linking and querying HCS metadata. Each strategy for storing metadata delivers a compromise between, on the one hand, strict typing and querying and on the other, data format flexibility. This allows developers and users to choose the approach that matches the requirements for their experiment.

**Fig. 1:**
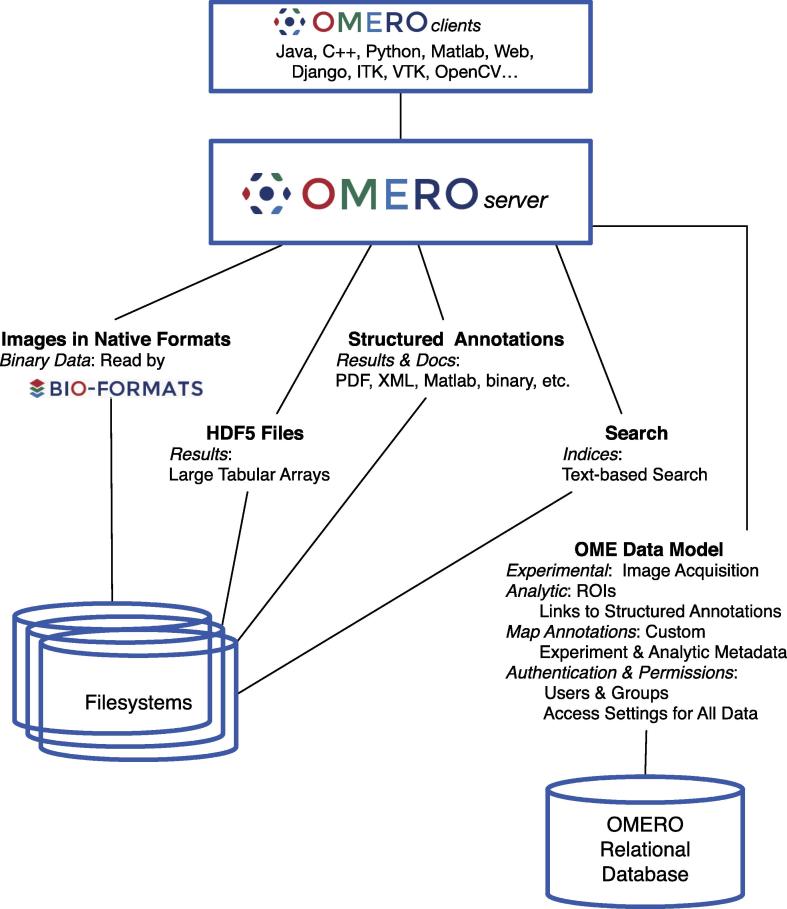
Metadata storage and retrieval in OMERO. The drawing shows the different types of metadata supported within OMERO and how they are stored. All these metadata are accessible in OMERO clients through the API presented by the OMERO server.

Fig. 1 summarizes the different data access and storage strategies available within OMERO. Different data types are stored in different ways, maximizing performance and flexibility. Regardless, all these data are accessible through the OMERO API, allowing a wide range of data analysis and visualization applications access to a wide range of data types and structures that comprise an HCS experiment. The different data types supported by OMERO are detailed in the following sections.

#### 3.3.1 Storing experimental metadata

Experimental descriptors like plate format, well position, and most common imaging parameters—fluorescence channel, exposure time, objective lens, and imaging detector—are all specified in the OME Data Model [8] and represented in OMERO’s database. Wherever possible, these metadata are read during import from proprietary image file formats using Bio-Formats and stored in OMERO’s database. They are then searchable and queryable through the OMERO API. These basic metadata are critical for properly recording an experiment, but usually miss essential experiment descriptors such as small molecules, siRNAs or other reagent details, and even essential details of the experimental protocol—cell types, incubation times and temperatures, etc. To support these metadata, we originally enabled custom extensions of the OME Data Model, supporting concepts like “Reagents”, etc. which we could not express in a completely generic way [8]. However, data model extensions require substantial technical expertise and are not accessible to most users. Therefore, starting in OMERO 5.1, we have introduced support for “map annotations”, or user-defined key-value pairs (e.g., “Temperature”:“37” or “Cell line”:“U2OS”). In addition, we extended the OME Data Model and the OMERO database to include support for scientific units, so that all quantities, including map annotations can be expressed in appropriate units (e.g., “Temperature”:“37”:“°C”; https://www.openmicroscopy.org/site/support/ome-model/developers/ome-units.html). Map annotations can be defined for metadata that are specific to each installation, OMERO group or user, and can therefore be used to define and record specific sets of metadata for an experiment or group. They are easily extensible, queryable from the OMERO API and the default OMERO Java and web clients provide support for displaying map annotations where they are available. As described below, we have created several scripts to load metadata stored as spreadsheet (i.e., tab delimited or CSV) files into OMERO as map annotations, making them accessible to most users.

#### 3.3.2 Storing analytic metadata

Analytic outputs are another form of metadata that must be supported in any HCS data management system. These include regions of interest (ROIs) that delineate objects such as cells and are often stored as masks or boundaries, and measurements such as intensities, areas, and spatial statistics. Features may be obtained at multiple scales ranging from single ROIs to aggregated images, and may be further processed to obtain a phenotypic label, with machine learning algorithms often used to identify interesting patterns in the data, perhaps in conjunction with external bioinformatics databases (for reviews, see [12], [13]). A final requirement is the storage and recall of the parameters used for any analysis algorithms, with the flexibility to accommodate differences in implementations between different scientists and laboratories, and the evolution of parameters used for different runs of any algorithm. An HCS data management system must support the storage and accurate linking of all these complex data and also include as much querying capabilities as possible.

OMERO provides two mechanisms to support storage of analytic parameters and outputs. The first is well-established, and amounts to a mechanism for storing analysis metadata files, regardless of format (e.g., .txt, .xls, .doc, .m, .pdf, etc. are all supported) as annotations on an image, plate or screen, as required by the analysis. These metadata files are given a defined namespace, making the files accessible for future download and linkage. No direct querying of metadata stored as annotations is supported, although text-based metadata is indexed by OMERO’s Lucene search engine, so a text-based search will return the files and their contents. This approach provides maximum flexibility, but provides only marginally more structure and query capability than a filesystem.

A more structured approach for storing analytic metadata, but with enough flexibility to support the great diversity in analytic outputs generated in HCS experiments is an HDF-based tabular data store, called OMERO.tables [3]. This data management mechanism targets large tabular arrays, like those generated in the analysis of cell-based HCS experiments. As an example, the analytic output from a single 384-well plate with 5 images/well, 3 channels/image, 50 cells/image and 25 calculated feature parameters/channel/cell requires a table with at least 53 columns (enough to represent the well, the image in the well, the ROIs and all features for all channels) and 7.2 million rows. In OMERO, metadata related to wells, images and ROIs can be stored in the OMERO database, but the feature parameters can be stored via the OMERO API in a tabular array stored in an HDF5 file, allowing fast writes and reads of large arrays (writing datasets like the 384-well example described above requires 10–15 s on standard hardware). The API supports writing of rows and naming of columns, allowing support for the different types of analytic metadata recorded in different assays. Each feature table row can be linked back to its source object by a unique ID. Recalling whole or parts of columns is supported by the API, but full SQL-like querying is not possible. The approach provides less structure and definition than a fully-typed database, but much better performance and more querying capabilities than a CSV file on a file system.

#### 3.3.3 Using OMERO for HCS metadata management

Given the scale of HCS experiments, experimental and analytic metadata will be entered into OMERO automatically, either during import of data, or during or after analysis runs. For data import, the OMERO.insight desktop Java client and the OMERO.cli command line importer use Bio-Formats to recognize and translate experimental metadata into OMERO. In cases where analytic metadata or large metadata collections are recorded in spreadsheets, customizable scripts can be used for loading into OMERO. They can be run at data import, or at a later time, to link analytic metadata that is calculated outside of OMERO with an HCS screen or other large dataset.

For analysis functions using Matlab, a fully developed Matlab API is available for reading from and writing to OMERO (see for example [5]). Python data analysis tools (e.g., http://pydata.org/) can work directly with the OMERO Python API. Results can be stored as file annotations, map annotations, or via the OMERO API in HDF5-based tabular stores.

### 3.4 HCS data repositories

There is increasing interest in including datasets and metadata alongside traditional scientific publication to support scientific integrity and, where appropriate, data reuse. This is particularly important for HCS data as the scale of the experiments provide opportunities for re-analysis and validation of published results. Published HCS datasets also provide benchmark datasets that support the development of new analysis tools by members of the scientific community. In these cases, linkage of experimental and analytic metadata is again critical, to ensure that anyone who accesses published HCS datasets can easily assess the protocols, acquired data and derived results without having to manually reconstruct the original linkages between metadata and binary image data.

At the time of this writing, there are several first generation HCS data repositories available. Data Dryad, a non-profit scientific data repository, hosts HCS datasets from published studies [14], [15], although these resources only make files available for download and do not provide any direct linkage of metadata and binary data. The ASCB Cell Image Library, an OMERO-based repository also holds images related to HCS screens, but again provides no explicit metadata linkage [16]. The Broad Benchmark Bioimage Collection provides a series of public, annotated images from screens from several species and provides metadata search [17]. The JCB DataViewer, another OMERO-based image data repository linked to the *Journal of Cell Biology*, has published nine genome-wide knockout or knockdown screens that include linkage and display of experimental and analytic metadata (http://jcb-dataviewer.rupress.org/?view=hcs). The Library of Integrated Cellular Signatures (LINCS) has used OMERO to publish the phenotypes of cell lines treated with a standard compound library [18]. These resources, along with several individual projects (see Table 1) all serve as examples of efforts to publish individual HCS studies. Two resources have been built that integrate data from more than one HCS dataset. Mitocheck (http://mitocheck.org) publishes several different screens and allows gene and phenotypic querying across them. A follow-on application, the Cellular Phenotype Database [19] integrates the Mitocheck datasets with several others and delivers a first attempt at systematic phenotypic search using a defined cell phenotype ontology, the Cell Microscopy Phenotype Ontology (http://www.ebi.ac.uk/cmpo/). The GenomeRNAi database provides access to experimental and analytic metadata for >400 screens, but publishes no images [20].

By analogy to early genome resources, these published datasets are valuable and serve the twin goals of validation and re-use. Moving forward, the data in these resources need to be combined with others, allowing HCS data aggregation and querying of results from studies across orthologous genes or classes of small molecules, and the exploration of genetic perturbations and/or small molecules that are linked to similar phenotypes. OMERO’s metadata storage and retrieval capabilities and broad support for many different image data formats and modalities make it an ideal platform for next-generation HCS data repositories. Image acquisition metadata are already supported by the OME Data Model; experimental metadata covering small molecule or genetic perturbations (e.g., siRNA, CRISPR/Cas9, etc) are well-suited to OMERO’s map annotations; analytic outputs including ROIs and features are supported by the OME Data Model and OMERO.tables. Currently, the OMERO API does not have explicit support for ontological annotations, but a map annotation declaring an ontology name and ID would provide sufficient information for a look-up of more detailed info and subsumption queries on the Ontology Lookup Service [21]. We are currently attempting to build such a resource, based on an aggregation of most of the datasets in Table 1.

## 4 Discussion

The size and complexity of HCS datasets requires enterprise-level software tools that can be deployed in labs and institutes, that can handle multi-terabyte file sets, and that support many different types of image data and metadata. We have built two open-source tools that provide foundations for enterprise HCS data management. Bio-Formats reads image data and metadata from >140 different file formats, making a large number of image files and modalities available in a common model. OMERO uses Bio-Formats and supports scaled data access and management via a single API. It is a client–server application that provides several different methods for storing metadata, ranging from strongly typed and queryable to more flexible and indexed for search. OMERO’s architecture recognises that there is not a single type of HCS experiment, and that different approaches and assays require different strategies for storing and managing image data and metadata.

The challenges of HCS data management extend past the datasets collected in any single laboratory or screening facility. OMERO includes support for defining data access for colleagues, so that data can be held privately, shared with a defined group, and even assigned to another user (in cases where data is acquired by one user and then handled and analysed by another). The logical extension of this data sharing capability is full on-line publication of specific datasets, which OMERO also supports. OMERO has already been used to publish several HCS datasets as individual entities (Table 1). A critical next step is enabling public querying across datasets, so that genes, small molecules and phenotypes can be systematically queried, and all components of any results, including the images and analytic outputs can be viewed. Looking forward, making public HCS datasets not only browseable and queryable but also accessible for re-analysis is a critical next step. This is important for ensuring that methods and conclusions can be validated and for testing and benchmarking. Most importantly, the spectrum of HCS experiments now publicly available represent a very small sampling of possible experimental manipulations and measured phenotypes. The similarity between phenotypes measured in a HeLa cell, a U2OS cell, an MCF10A cell, a Drosophila S2 cell, any number of human iPS cells or an S. pombe cell caused by either gene product knockdown or small molecule inhibitor is not known. An understanding of the basis for cell and tissue phenotypes in HCS experiments, and thus the basis for genetic and therapeutic effects in living organisms will be the outcome of properly constructed, well-populated public HCS databases.

## Figures and Tables

**Table 1:** Published HCS datasets. A list of HCS studies that have published full datasets—metadata and binary image data—for browsing, query and potential download.

Study	Cell line/organism	Phenotypes measured	Perturbations	Dataset resource(s)	References
Mitocheck	Human HeLa	Cell division defects	Genome-wide siRNA	http://mitocheck.org	[22]
Yeast proteome plasticity	*S. cerevisiae*	Stress-based protein localization changes	Oxidative stress; starvation	http://jcb-dataviewer.rupress.org/jcb/browse/6203/	[23]
Nuclear body components	Human HeLa	Nuclear body localization	Genome-wide siRNA; ORFeome 5.1	http://jcb-dataviewer.rupress.org/jcb/browse/6852/S152/	[24]
Cell–cell adhesion	Drosophila S2	Adherent cells	Primary genome-wide siRNA	http://jcb-dataviewer.rupress.org/jcb/browse/7555/S202/	[25]
Cell–cell adhesion	Canine MDCK	Adherent cells	Secondary siRNA	http://jcb-dataviewer.rupress.org/jcb/browse/7555/S252/	[25]
SUMO function	*S. cerevisiae*	Nuclear & cytoplasmic phenotypes	Non-essential mutant library	http://jcb-dataviewer.rupress.org/jcb/browse/6156/S52/	[26]
DNA damage response	*S. cerevisiae*	Rad52 localisation	Non-essential mutant library	http://jcb-dataviewer.rupress.org/jcb/browse/4608/S1/	[27]
Cytoskeletal structure	Drosophila S2	Actin, microtubule localization	Primary genome-wide siRNA	http://jcb-dataviewer.rupress.org/jcb/browse/4609/S2/	[28]
Cytoskeletal structure	Human Hela	Actin, microtubule localization	Secondary siRNA	http://jcb-dataviewer.rupress.org/jcb/browse/4609/S3/; http://jcb-dataviewer.rupress.org/jcb/browse/4609/S4/	[28]
Sysgro	*S. pombe*	Cell shape, microtubule defects	Non-essential mutant library	http://sysgro.org	[29]
SH4 Protein targeting	Human HeLa	SH4 domain membrane targeting	Genome-wide siRNA	http://www.ebi.ac.uk/fg/sym/study/B1_SyM	[30]
DNA damage response	Human HeLa; U2OS	53BP1 foci formation	Genome-wide siRNA	http://mitocheck.org/cgi-bin/mtc; http://www.ebi.ac.uk/fg/sym/study/C2_SyM	[31]
ER->Plasma membrane secretion	Human HeLa	tsO45G localization	Genome-wide siRNA	http://mitocheck.org/cgi-bin/mtc; http://www.ebi.ac.uk/fg/sym/study/E1_SyM	[32]
Systems survey of endocytosis	Human HeLa	Transferrin & EGF endocytosis	Genome-wide siRNA/esiRNA	http://endosomics.mpi-cbg.de/	[33]
DNA damage-induced histone ubquintinylation	Human U2OS	GFP-RNF168 localisation to damage loci	Genome-wide siRNA	http://mitocheck.org/cgi-bin/mtc; http://www.ebi.ac.uk/fg/sym/study/G1_SyM	[34]
LINCS	Human various	Apoptosis, proliferation	Mitotic & mTOR inhibitors	http://lincs.hms.harvard.edu/db/	[18]
Broad Bioimage Benchmark	Various	Various	Mutant and siRNA screens	https://www.broadinstitute.org/bbbc/	[17]
DNA damage response	Human Mac2a, K299	Chromosome breaks, translocations	hiBA-FISH; probes that reveal chromosome breaks	http://dx.doi.org/10.5061/dryad.6h7nt	[14]
Cell painting	Human U2OS	Cell phenotype marker localisation	30,000 compounds; various sources	http://www.cellimagelibrary.org/pages/project_20269	[16]
